# LncRNA SLC16A1‐AS1 contributes to the progression of hepatocellular carcinoma cells by modulating miR‐411/MITD1 axis

**DOI:** 10.1002/jcla.24344

**Published:** 2022-03-15

**Authors:** Chun Duan

**Affiliations:** ^1^ Department of Infectious Diseases Yijishan Hospital of Wannan Medical College Wuhu China

**Keywords:** hepatocellular carcinoma, metastasis, miR‐411, MITD1, SLC16A1‐AS1, tumorigenesis

## Abstract

**Background:**

Hepatocellular carcinoma (HCC) is a common malignancy with high morbidity. The current study aimed to explore the molecular mechanism of lncRNA SLC16A1‐AS1 in the tumorigenesis of HCC.

**Material and methods:**

The expression of SLC16A1‐AS1 and miR‐411 was examined in clinical HCC tissues. HCC cell lines Hep3B and Huh‐7 were employed and transfected with si‐SLC16A1‐AS1. The correlation between SLC16A1‐AS1 and miR‐411 was verified by luciferase reporter assay. Cell viability was detected by CCK‐8 assay. Cell migration and invasion capacity were examined by transwell assay. The protein level of MITD1 was analyzed by western blotting.

**Results:**

The expression of SLC16A1‐AS1 markedly increased in HCC tissues and cell lines. Subsequent studies identified SLC16A1‐AS1 as a downstream target of miR‐411. In addition, SLC16A1‐AS1 knockdown and miR‐411 overexpression significantly stagnated the progression of HCC cells. SLC16A1‐AS1 knockdown also downregulated MITD1 levels.

**Conclusion:**

Our findings showed that SLC16A1‐AS1 was overexpressed in HCC cells and tissues. SLC16A1‐AS1 promoted the malignant characteristics of HCC cells and acted as an oncogene. Its regulatory effect may be associated with miR‐411/MITD1 axis. Therefore, SLC16A1‐AS1 has the potential to be used as a biomarker or therapeutic target for the treatment of HCC.

## Graphical abstract

Hepatocellular carcinoma (HCC) is a common malignancy with high morbidity. The current study aimed to explore the molecular mechanism of lncRNA SLC16A1‐AS1 in the tumorigenesis of HCC, and found SLC16A1‐AS1 is upregulated in HCC cells and tissues.

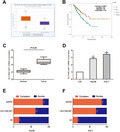

## 1 INTRODUCTION

Hepatocellular carcinoma (HCC) is the fifth most common cancer in the world, and male mortality ranks second, especially in Africa and Asia.^1^ Although the treatment of HCC has made progress in recent decades, HCC remains the third leading cause of cancer‐related deaths worldwide. In approximately 90% of cases, HCC is related to liver cirrhosis.^2^ One‐third of patients with liver cirrhosis will develop HCC in the future.^3^ Liver cirrhosis is an advanced scar formation process. Due to long‐term liver injury, healthy liver tissue is replaced by nodules and scar tissue, and the scar tissue is surrounded by fiber bundles.^4^ Irreversible liver cirrhosis may eventually develop into HCC. Therefore, finding new targets for therapeutic and diagnostic methods will help in the early diagnosis and intervention of HCC.

Studies have shown that less than 1.5% of the human genome has a protein‐coding function, and some of the rest contain functionally conserved non‐coding series.^5^ A large part of the genome is transcribed into non‐coding RNAs (ncRNAs), with no or low protein‐coding functions. NcRNA can be divided into several families according to its molecular size.^6^ LncRNA is defined as a transcript with a length of more than 200 nucleotides.^7^ Recently, a lot of attention has been focused on lncRNA. With the development of genome‐wide transcriptomics analysis, lncRNA has been confirmed to participate in a variety of biological activities, such as cell cycle regulation, apoptosis, differentiation, and other life activities.^8^, ^9^ The latest research shows that lncRNA can affect HCC metastasis‐related molecules at the transcription level and post‐transcription level, and provide potential diagnostic indicators and therapeutic targets for HCC patients.^10^

The current study aims to better understand the molecular mechanism of SLC16A1‐AS1 in the occurrence and development of HCC. The expression level of SLC16A1‐AS1 in clinical specimens and cells was determined. Function assay uncovered the role of SLC16A1‐AS1 in the malignant phenotypes of HCC cells. Further study investigated the molecular interaction between SLC16A1‐AS1 and miR‐411/MITD1 axis. This article analyzes in detail the role of SLC16A1‐AS1 in the progression of HCC, which will help to understand the pathogenesis of HCC.

## 2 MATERIALS AND METHODS

### 2.1 Clinical specimens

The present study was conducted with the permission of the Ethics Committee of Wannan Medical College and carried out according to the guidelines of ethical management. All participants provided written informed consent. A total of 30 HCC specimens and paired normal tissues were obtained from Yijishan Hospital of Wannan Medical College from 2019 to 2021. The including criteria were as follows: (1) all patients pathologically diagnosed with primary HCC, and diagnosis was based on the World Health Organization criteria; (2) no surgical treatment, radiotherapy, chemotherapy, and biotherapy were performed before operation; (3) complete clinical records and follow‐up information were available in all cases. Patients were excluded if any of the following conditions were met: (1) presence of cancers other than HCC; (2) autoimmune hepatitis or toxic hepatitis; (3) refusal to participate.

### 2.2 Cell culture and reagents

The human HCC cell lines (Hep3B and Huh‐7) and human normal hepatic cell line (LO2) were purchased from ATCC (American Type Culture Collection). The cells were cultured in DMEM medium supplemented with 10% fetal bovine serum (GIBCO). SiRNA‐SLC16A1‐AS1 and negative control miRNA were synthesized by Biossci Company. Transfections were performed using Lipofectamine 2000 (Invitrogen).

### 2.3 Quantitative real‐time PCR

The total RNA was isolated from cells and tissues using Trizol reagent (Life Technologies). First‐strand cDNA was carried out using reverse transcription reagents (ABI). Quantitative real‐time PCR (qRT‐PCR) was performed using SYBRH Select Master Mix for CFX (Invitrogen) and using the CFX Connet TM real‐time PCR system (Bio‐Rad). GAPDH and U6 were chosen as the internal standard. The primer sequences used are shown in Table 1.

**TABLE 1:** RT‐PCR primer sequences

Gene	Primer sequence (5′−3′)
SLC16A1‐AS1	F: GCCAACGTTAGGCCCAAATTACGA R: GGCTTAATCTGCTTGACGTCTTGC
miR‐411	F: CCATGUAUGUAACACGGUCCAC R: UAGCGCUGGACCGGTCTCA
MITD1	F: AGCTCACGGAGTGTGGTTCAACT R: CGACTGGGCAACATGGTCGCGATC
U6	F: TCGCTTTCGGCAGCACATATAC R: AACGCTTCACGAGCGTGTCTGTC
GAPDH	F: TACAGCAAAGGACTCGTGGA R: TCGACCATGCCAGTGAGCATTC

### 2.4 Cell viability assay

Cell viability was measured using Cell Counting Kit‐8 assay (Beyotime). After being inoculated into 96‐well plates at a density of 2 × 10^3^ cells/well, cells were stained with 20 μl of CCK8 reagent 48 h after transfection. Cell viability was determined at the 450 nm absorbance.

### 2.5 Invasion assay

A two‐chamber Transwell assay determined the invasion of target cells (Corning Incorporation). Target cells were suspended in a 200 µl serum‐free medium and added to the upper chamber precoated with Matrigel (500 ng/µl; BD Biosciences). Complete medium (600 µl) was added to the lower chamber. After incubation for 48 h at 37°C, cells invading the lower chamber were immediately fixed with methanol. Non‐invasive cells in the upper chamber were carefully removed using cotton swabs, while invasive cells in the lower chamber were stained with crystal violet and counted under a microscope. Cell invasion (%) was calculated as the average invasive cells in the experimental group/average migrated cells in the control group × 100%.

### 2.6 Wound healing assay

Cells were seeded in 6‐well plates. After 12 h of incubation, wounds were generated with a 200 μl pipette tip. Cells were washed and incubated with serum‐free medium for 48 h. The wounded gap was photographed at different time points. Cell migration was calculated as wound closure (%) = (wounded area (A0‐A1)/wounded area A0) × 100%.

### 2.7 Dual‐luciferase assay

The psi‐CHECK‐2 psi‐check Luciferase Expression Reporter (Promega) vector was used to synthesize SLC16A1‐AS1 vector. Hep3B and Huh‐7 cells were used for the dual‐luciferase reporter assay. Luciferase activity was measured and compared 48 h later. Luciferase assay was performed 48 h after transfection using a Dual‐Luciferase Reporter Assay System (Promega).

### 2.8 Western blot

Cells were washed twice with PBS, and total protein was extracted using RIPA buffer (Beyotime Biotechnology). Protein concentration was determined using a BCA Protein Assay Kit (Beyond Biotechnology). Protein samples were separated using 10% SDS‐polyacrylamide gel (Solarbio) after denaturation. Then, proteins were transferred to a PVDF membrane (Millipore) which were blocked with 5% skimmed milk for 1 h. The blots were incubated overnight with the first antibodies (anti MITD1 antibody, 1:5000, Santa Cruz; anti‐GAPDH antibody, 1:5000; Santa Cruz). ECL (Millipore) After incubation with secondary antibodies, an enhanced chemiluminescence reagent was applied for detection.

### 2.9 Statistical analysis

All statistical analyses used SPSS v22.0 (SPSS Inc.). Each experiment was repeated at least three times. Data were shown as mean ± standard deviation. The significance of differences between groups were assessed by Student's t test and ANOVA test. *p*‐Values <0.05 were considered statistically significant.

## 3 RESULTS

### 3.1 SLC16A1‐AS1 is upregulated in HCC cells and tissues

**FIGURE 1:**
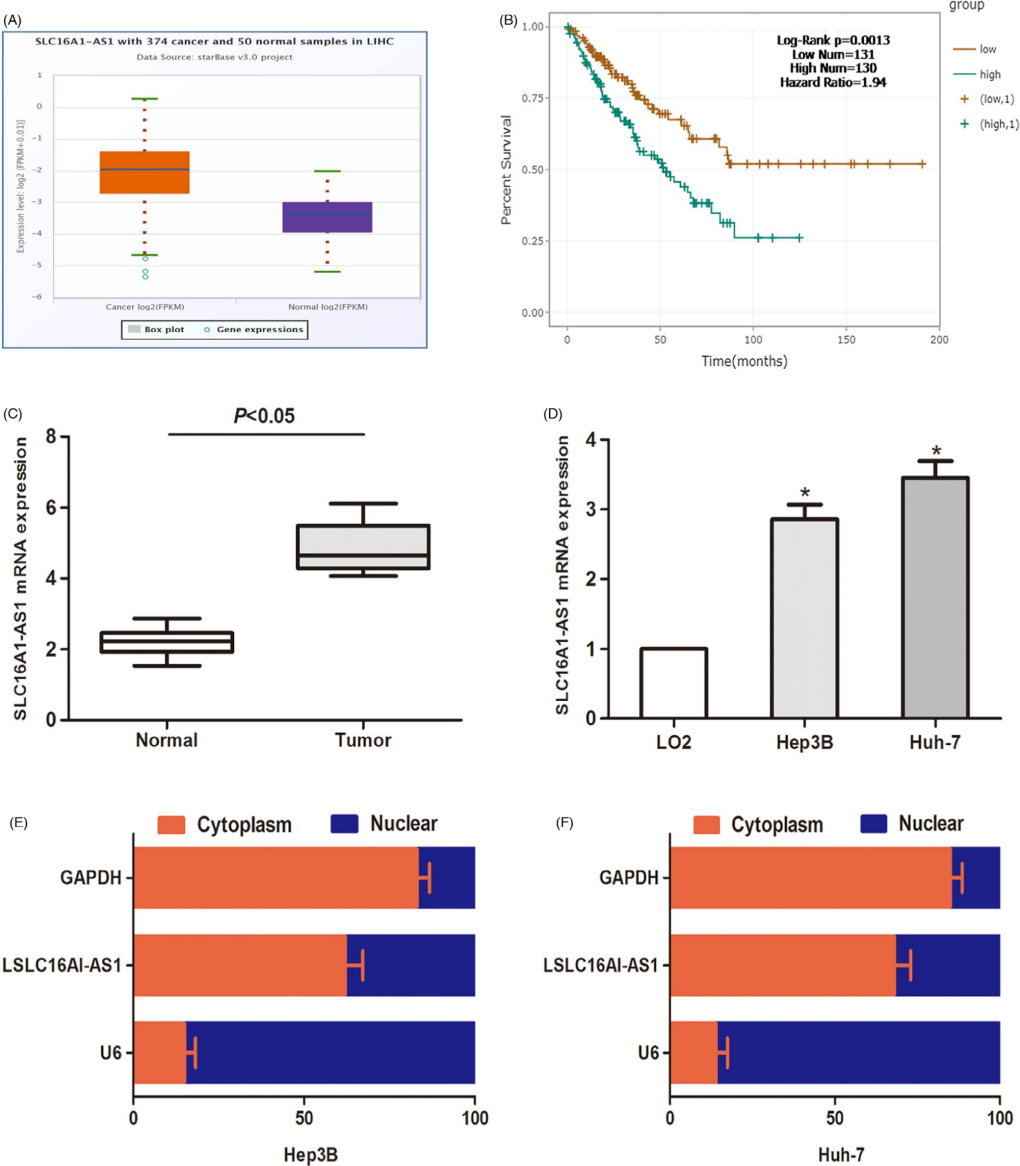
SLC16A1‐AS1 is upregulated in hepatocellular carcinoma (HCC) cells and tissues. (A) Data from Starbase 3.0 show the expression of LncRNA SLC16A1‐AS1 in HCC tissues and normal tissues. (B) Survival analysis of SLC16A1‐AS1 in patients with HCC. (C) SLC16A1‐AS1 expression in HCC tissues and normal tissues. (D) SLC16A1‐AS1 expression in HCC cells (Hep3B and Huh‐7) and human normal hepatic cell line (LO2). (E and F) The distribution of SLC16A1‐AS1 in the subcellular fractions of Hep3B and Huh‐7 cells. U6 and GAPDH were used as nuclear and cytoplasmic markers, respectively. (*p < 0.05)

To determine the expression of SLC16A1‐AS1 in HCC tissues, we explored the expression of SLC16A1‐AS1 in the TCGA data portal of starbasever3.0. As shown in Figure 1A, compared to normal tissues, SLC16A1‐AS1 increased significantly in HCC tissues. Accordingly, the expression of SLC16A1‐AS1 in HCC tissues and cells was significantly higher than that in normal cells (Figure 1C,D). Kaplan–Meier analysis was performed, and the data revealed that compared to patients with low SLC16A1‐AS1, patients with high SLC16A1‐AS1 had a worse overall survival time (Figure 1B). In addition, the subcellular distribution experiment was used to study the expression area of SLC16A1‐AS1 in HCC cells. The results showed that SLC16A1‐AS1 was mainly expressed in the nucleus rather than the cytoplasm (Figure 1E,F). In summary, our findings suggest that SLC16A1‐AS1 is overexpressed in HCC.

### 3.2 SLC16A1‐AS1 regulates proliferation and invasion of HCC in vitro

**FIGURE 2:**
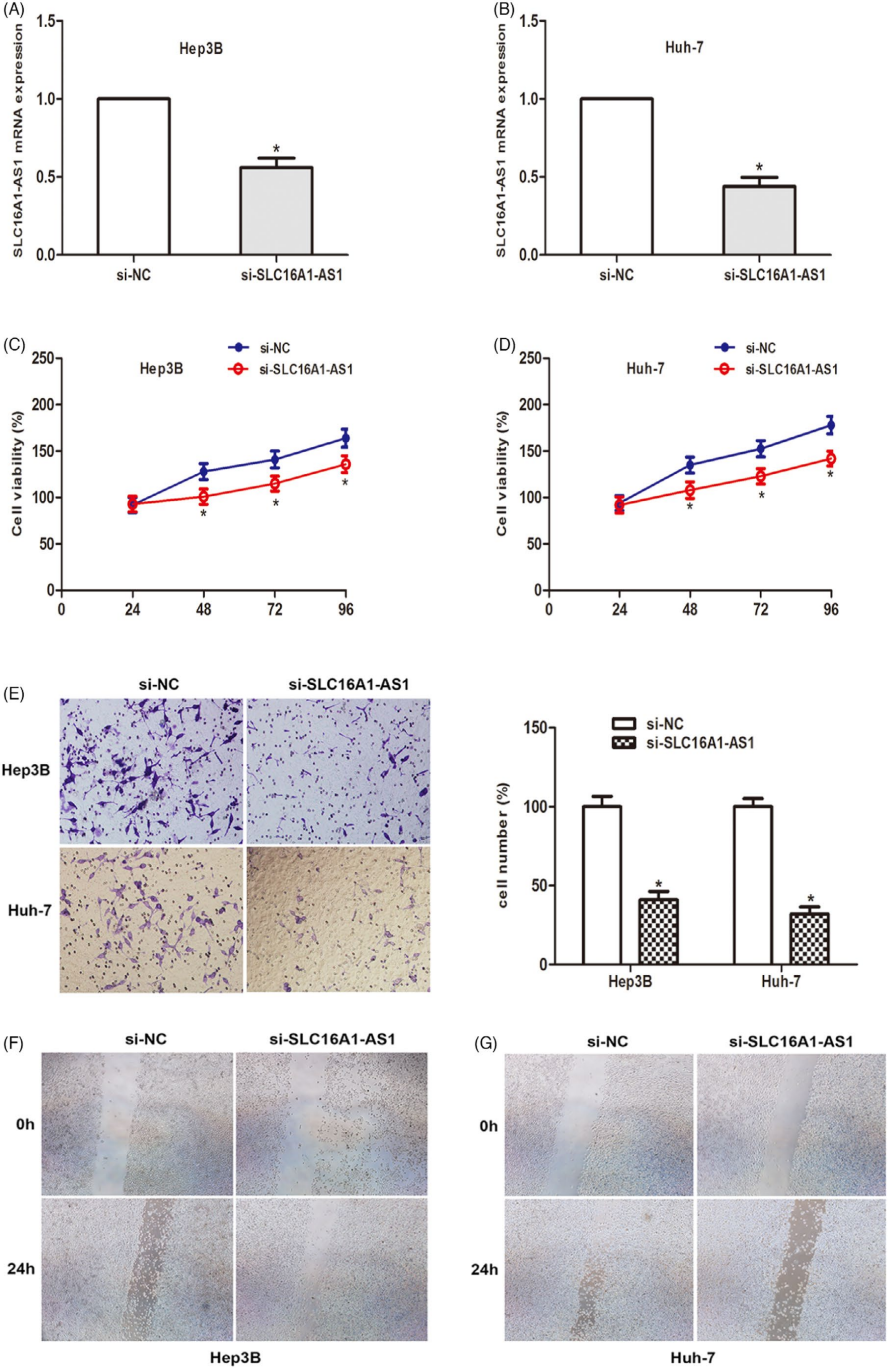
SLC16A1‐AS1 regulates proliferation and invasion of hepatocellular carcinoma (HCC) in vitro. (A and B) qRT‐PCR results showed si‐SLC16A1‐AS1 were transfected into Hep3B and Huh‐7 cells successfully. (C and D) CCK‐8 assay was performed to determine cell viability of cells transfected with si‐SLC16A1‐AS1. (E) Transwell invasion assay was performed to investigate invasive capacity of cells transfected with si‐SLC16A1‐AS1. (F and G) Wound healing assay was performed to evaluate the migratory level. (*p < 0.05)

Considering the increased expression of SLC16A1‐AS1 in HCC, we explored the effect of SLC16A1‐AS1 gene knockdown on the malignant phenotype of HCC cells. By transfection of siRNA, the expression level of SLC16A1‐AS1 in Hep3B and Huh‐7 cells was significantly reduced (Figure 2A,B). Then CCK‐8 analysis was performed, and the results showed that SLC16A1‐AS1 knockdown significantly inhibited the proliferation of the two HCC cell lines (Figure 2C,D). At the same time, the transwell invasion test was used to detect the invasion ability of the cells. The data showed that compared to the NC group, the number of invasive cells in the si‐SLC16A1‐AS1 group was significantly reduced (Figure 2E). Cell migration was measured by wound healing experiments. The data showed that SLC16A1‐AS1 silencing significantly inhibited cell migration (Figure 2F,G). The above results suggest that SLC16A1‐AS1 gene knockdown can inhibit the proliferation and invasion of HCC cells.

### 3.3 SLC16A1‐AS1 acts as a ceRNA in HCC cells

**FIGURE 3:**
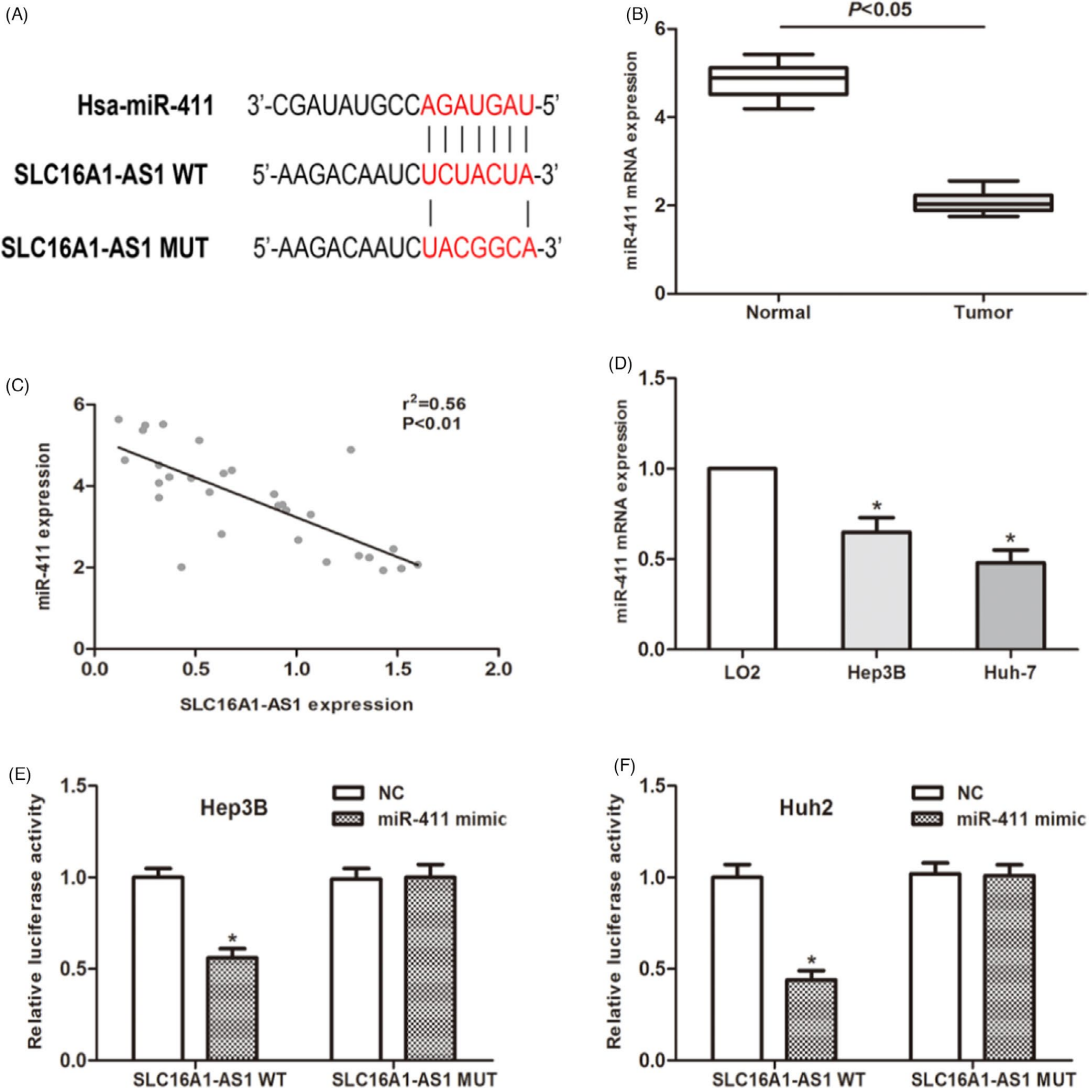
SLC16A1‐AS1 acts as a ceRNA in hepatocellular carcinoma (HCC) cells. (A) Putative binding site of miR‐411 on SLC16A1‐AS1. (B) MiR‐411 expression in HCC tissues and normal adjacent tissues was measured using qRT‐PCR. (C) Pearson's correlation analysis was performed to determine the relationship between miR‐411 and SLC16A1‐AS1. (D) MiR‐411 expression in HCC cells was detected by qRT‐PCR. (E and F) Dual‐luciferase reporter assay was performed to determine luciferase activity in cells co‐transfected with miR‐411 mimic and SLC16A1‐AS1‐WT or SLC16A1‐AS1‐MUT. (*p < 0.05)

It has been confirmed that lncRNA regulates the expression of miRNA through the sponge effect. Therefore, we used the TargetScan database to identify candidate miRNAs that interact with SLC16A1‐AS1. The potential binding site of miR‐411 is shown in Figure 3A. QRT‐PCR analysis suggested that miR‐411 is lower expressed in HCC tissues compared to paired normal tissues (Figure 3B). Then, a Pearson's correlation analysis was performed. Our data suggested that miR‐411 expression level was negatively associated with the expression of SLC16A1‐AS1 in 30 HCC patients (Figure 3C). The expression level of miR‐411 in HCC cells was also detected and found that its expression level was downregulated as expected (Figure 3D). To further verify our hypothesis, we performed a luciferase reporter analysis to explore the correlation between miR‐411 and SLC16A1‐AS1. The results showed that miR‐411 significantly inhibited the luciferase activity of the cells transfected with SLC16A1‐AS1‐Wt, while the luciferase activity of the SLC16A1‐AS1‐Mut group was not affected (Figure 3E,F). As mentioned above, the above data indicate that SLC16A1‐AS1 acts as a ceRNA to regulate the expression of miR‐411 in HCC cells.

### 3.4 MiR‐411 regulates malignant phenotypes of HCC cell

**FIGURE 4:**
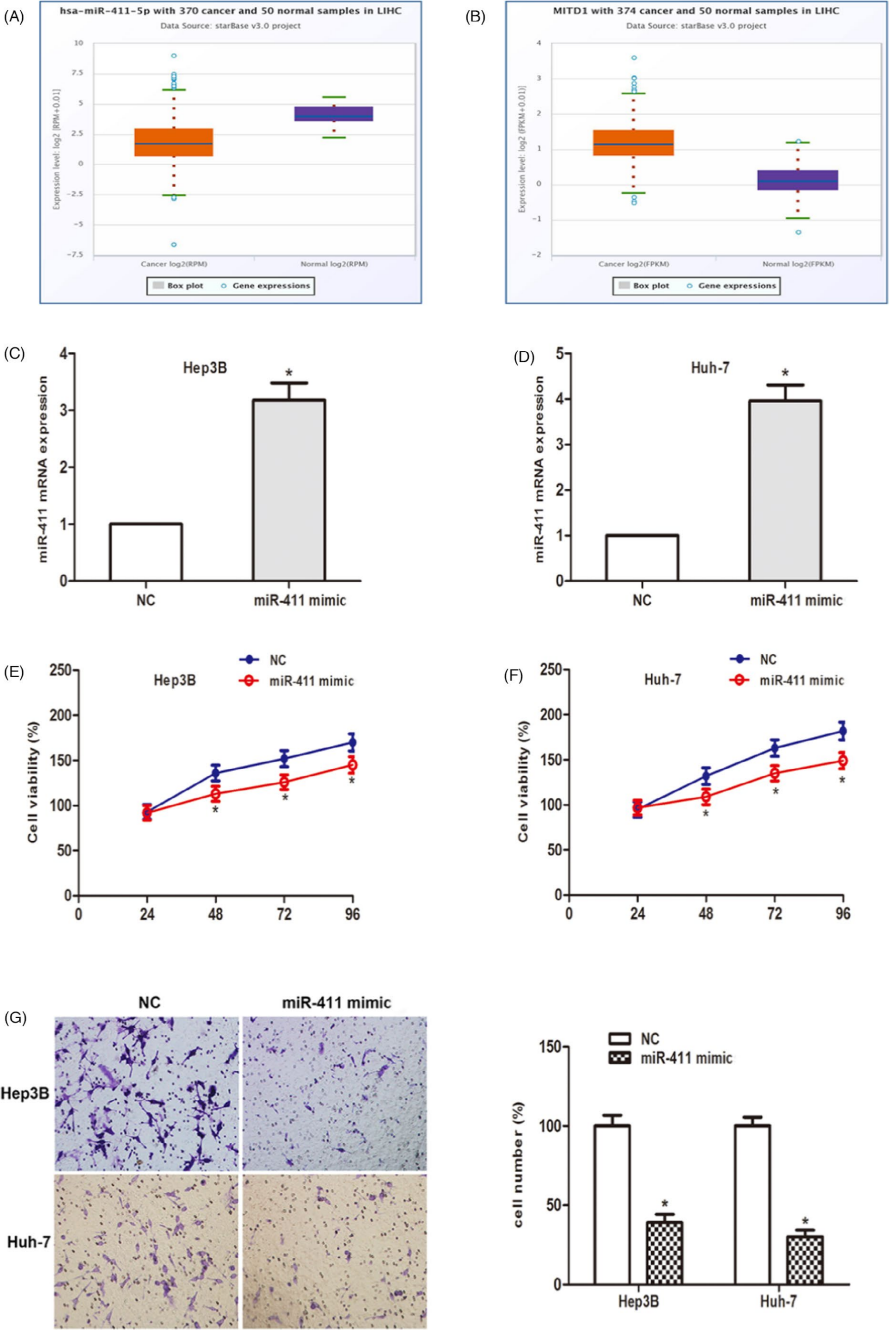
MiR‐411 regulates malignant phenotypes of hepatocellular carcinoma (HCC) cell. (A and B) Data from Starbase 3.0 showed the expression of miR‐411 and MITD1 in HCC tissues and normal tissues. (C and D) qRT‐PCR results showed miR‐411 mimic were transfected into Hep3B and Huh‐7 cells successfully. (E and F) CCK‐8 assay was performed to detect cell viability of cells transfected with miR‐411 mimic. (G) Transwell invasion assay was performed to investigate cell invasive ability. (*p < 0.05)

The TCGA data portal of starbasever3.0. As shown in Figure 4A,B, compared to normal tissues, miR‐411 decreased and MITD1 increased significantly in HCC tissues. To study the biological functions of miR‐411 in vitro. After transfection with miR‐411 mimic, the expression of miR‐411 in HCC cells was detected. The data showed that miR‐411 was significantly increased in HCC cells (Figure 4C,D). Cell viability assays showed that miR‐411 overexpression remarkedly stagnated cell proliferation (Figure 4E,F). Then the cell invasion experiment showed that in the two HCC cell lines, the number of invaded cells was obviously reduced after the miR‐411 mimic was transfected compared to the miR‐411 mimic NC (Figure 4G). In summary, these findings suggest that miR‐411 plays a tumor suppressor role in the proliferation and invasion of HCC cells.

### 3.5 MiR‐411 directly targets MITD1

**FIGURE 5:**
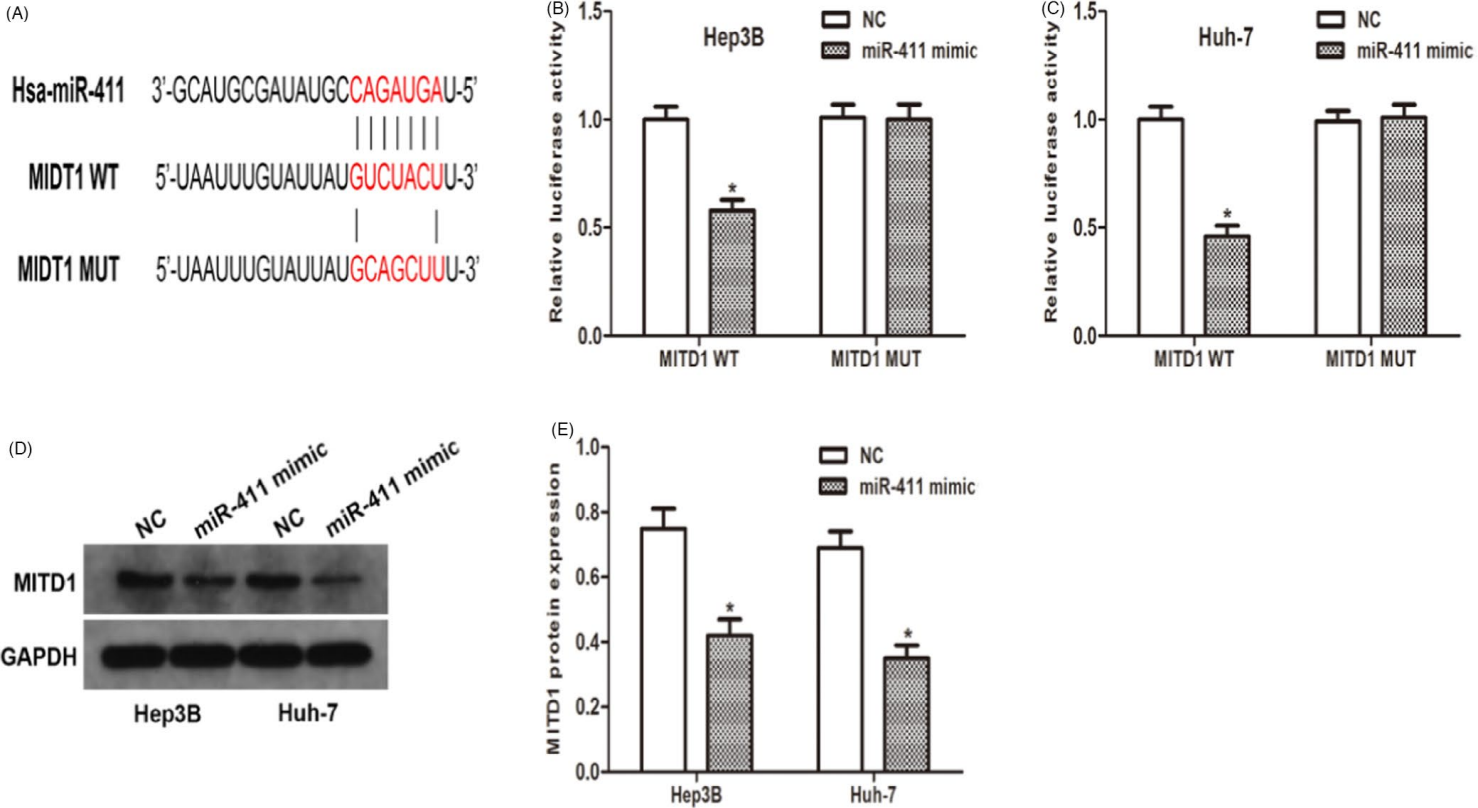
MiR‐411 directly targets MITD1. (A) Sequence alignment of predicted miR‐411 binding sites with the MITD1 3′UTR and the mutated sequence of miR‐411. (B and C) Luciferase reporter assay was performed in hepatocellular carcinoma (HCC) cells that were co‐transfected with miR‐411 mimic and reporter vectors that contain MITD1 3′UTR or mutated MITD1 3′UTR. (D and E) Western blot analyses showed that overexpression of miR‐411 markedly decreases the MITD1 expression level in HCC cells. (*p < 0.05)

With the help of open access databases (TargetScan, MiRanda and Starbase3.0), we determined that MITD1 is a potential downstream target of miR‐411. Therefore, a dual‐luciferase reporter plasmid containing WT or MITD1 3′UTR mutants was synthesized (Figure 5A). The plasmid containing MITD1 3′UTR was co‐transfected with miR‐411 mimic or NC mimic into Hep3B and Huh‐7 cells. Determination of luciferase activity. The data from the dual‐luciferase reporter gene analysis showed that the 3′UTR mutation of MITD1 abolished the inhibitory ability of miR‐411 compared to the wild type (Figure 5B,C). In addition, miR‐411 overexpression significantly reduced MITD1 protein levels (Figure 5D,E). In conclusion, our results confirm that miR‐411 directly regulates MITD1 expression.

## 4 DISCUSSION

Numerous evidences showed that lncRNAs regulate a variety of biological processes and play an important role in tumorigenesis and progression. Recent studies have shown that dysregulated lncRNAs can be used as effective therapeutic targets for HCC. At present, some lncRNAs have been found to have carcinogenic effects. For example, lncRNA SUMO1P3, SNHG9, XIST were found to be upregulated in HCC and acted as oncogene.^11^, ^12^, ^13^ These findings indicated that lncRNAs have great potential to be used as therapeutic targets in HCC treatment. In our present study, we analyzed the expression of SLC16A1‐AS1 in HCC tissues and cell lines. SLC16A1‐AS1 was found to be markedly upregulated both in tissues and cells. Then, we conducted a functional study to explore the role of SLC16A1‐AS1 in HCC cells. SLC16A1‐AS1 silencing significantly suppressed cell proliferation, colony formation, and invasion of HCC cells. Collectively, the results verified that SLC16A1‐AS1 may act as an oncogene in HCC cells.

Previous studies have shown that lncRNAs may exert the ability to function as a miRNA sponge, competing for miRNA, and modulate the expression of miRNA. For instance, LncRNA XIST promotes HBV‐related HCC by upregulating TRIM25.^13^ LncRNA Lnc‐APUE promotes tumor growth by regulating miR‐20b/E2F1 axis in HCC.^14^ LncRNA SMASR inhibits lung cancer progression by negatively regulating TGF‐β/Smad signaling.^15^ These studies enlightened us to explore the potential miRNAs that interact with SLC16A1‐AS1. Using open access database prediction, we chose miR‐411 for further study. Further study confirmed the role of miR‐411 in HCC and the regulatory relationship between SLC16A1‐AS1 and miR‐411.

MITD1 encodes a protein that regulates the activity of ESCRT‐III and is necessary for normal cytokinesis. MITD1 has been shown to be involved in the shedding phase of cytokinesis.^16^ Disorders of shedding may lead to tumorigenesis and genetic instability, especially when combined with mitotic stress caused by oncogenes.^17^, ^18^ In our study, we identified MITD1 as a downstream target of miR‐411. SLC16A1‐AS1 regulated MITD1 expression by interacting with miR‐411. These data suggested that SLC16A1‐AS1 exerts its function through the miR‐411/MITD1 axis.

## 5 CONCLUSION

In conclusion, our study shows that SLC16A1‐AS1 is upregulated in HCC tissues and cells. SLC16A1‐AS1, as an oncogene in HCC, exerts a carcinogenic effect by regulating the miR‐411/MITD1 axis. Our research helps to understand the role of SLC16A1‐AS1 in HCC, suggesting that SLC16A1‐AS1 may be used as a target for liver cancer treatment.

## CONFLICT OF INTEREST

All authors declare no conflicts of interest.

## CONSENT TO PARTICIPATE

Prior written consent was well informed and signed by all participants.

## CONSENT FOR PUBLICATION

All patients signed consent for publication.

## Data Availability

The datasets used and analyzed during the current study are available from the corresponding author on reasonable request.
